# Lists2Networks: Integrated analysis of gene/protein lists

**DOI:** 10.1186/1471-2105-11-87

**Published:** 2010-02-12

**Authors:** Alexander Lachmann, Avi Ma'ayan

**Affiliations:** 1 Department of Pharmacology and Systems Therapeutics, Systems Biology Center New York (SBCNY), Mount Sinai School of Medicine, 1425 Madison Avenue, New York, NY 10029, USA

## Abstract

**Background:**

Systems biologists are faced with the difficultly of analyzing results from large-scale studies that profile the activity of many genes, RNAs and proteins, applied in different experiments, under different conditions, and reported in different publications. To address this challenge it is desirable to compare the results from different related studies such as mRNA expression microarrays, genome-wide ChIP-X, RNAi screens, proteomics and phosphoproteomics experiments in a coherent global framework. In addition, linking high-content multilayered experimental results with prior biological knowledge can be useful for identifying functional themes and form novel hypotheses.

**Results:**

We present Lists2Networks, a web-based system that allows users to upload lists of mammalian genes/proteins onto a server-based program for integrated analysis. The system includes web-based tools to manipulate lists with different set operations, to expand lists using existing mammalian networks of protein-protein interactions, co-expression correlation, or background knowledge co-annotation correlation, as well as to apply gene-list enrichment analyses against many gene-list libraries of prior biological knowledge such as pathways, gene ontology terms, kinase-substrate, microRNA-mRAN, and protein-protein interactions, metabolites, and protein domains. Such analyses can be applied to several lists at once against many prior knowledge libraries of gene-lists associated with specific annotations. The system also contains features that allow users to export networks and share lists with other users of the system.

**Conclusions:**

Lists2Networks is a user friendly web-based software system expected to significantly ease the computational analysis process for experimental systems biologists employing high-throughput experiments at multiple layers of regulation. The system is freely available at http://www.lists2networks.org.

## Background

Experimental biologists who incorporate high-content profiling experiments within their research often face the difficultly of understanding results from many different experiments, under different conditions, and at different layers of regulation. Results from such experiments report the activity of groups of genes that function together to give rise to changes in cellular phenotype. It is often desired to compare the results from studies such as mRNA expression microarrays, ChIP-chip or ChIP-seq (ChIP-X), RNAi screens, proteomics and phosphoproteomics in one coherent global framework. Several advanced data mining techniques have been developed to address the challenge of analyzing the complexity of such datasets. Approaches fall into different categories which include: network reconstruction, gene-set enrichment analyses, and dynamical modeling [1]. Gene-set enrichment analyses are probably the most practical and successful approach so far. With this approach groups of genes identified experimentally are associated with prior biological knowledge to identify overlap similarity with sets of annotated genes for the purpose of suggesting functional biological themes [2,3]. The usefulness of such analyses was demonstrated first in what is now a classical study by Mootha et al. [4] who examined changes in gene expression in muscle biopsies from diabetic patients to discover a group of genes associated with oxidative phosphorylation that are coordinately down regulated in diabetic muscles. Their study led to the development of the Gene Set Enrichment Analysis (GSEA) software and the development of the MSigDB database, a database containing over 5,000 lists of mammalian gene-sets where each gene-set is associated with a specific experiment or a specific common biological function used to label each gene-set [5].

A recent review by Huang, Sherman and Lempicki [3] enlisted 68 bioinformatics tools that perform enrichment analyses on lists of genes. Most of these tools use the Gene Ontology (GO) database [6] as the only source for gene-sets with specific functional category. However, several other enrichment tools are capable of linking gene lists with other annotated gene-sets, i.e., pathway databases such as KEGG [7], BioCarta, and GenMAPP [8]. Systems such as GSEA [5], DAVID [9], GFINDer [10], WebGestalt [11] are similar to the software Lists2Networks (L2N) we present here in a sense that they go beyond Gene Ontology enrichment analysis to integrate other types of biological prior knowledge. For example, DAVID, which started as a Gene Ontology enrichment tool, now provides enrichment analysis capabilities to examine lists of genes for enrichment in pathways, structural domains, protein interactions, disease association, tissue expression and more [9]. Another important feature that is implemented in some of these systems is the visualization of the overlap among lists. For example, BioVenn is a web-based tool that takes as input two or three lists and draws Venn diagrams showing the overlap among lists of genes [12]. Another feature that is only provided by few systems is the ability to manipulate and merge lists. Such functionality exists, for example, in WebGestalt which allows users to apply set operations such as union, complementation, and intersection on loaded lists [11].

All gene-set enrichment tools compute statistics of whether the overlap among two sets of genes is significant. This is an important feature since it is possible for two lists of genes to have some overlap but such overlap can be due to chance. Computing the statistical significance of overlap among pairs of gene-set lists can be achieved using different contingency table statistics such as Fisher exact, binomial proportions, or Chi-squared tests. These tests compute the probability of finding the same genes in two or more experiments, or in two or more functional categories. The Fisher exact test computes this statistics exactly whereas the binomial proportions or Chi-squared tests are approximations. The goal of applying these tests is to find gene-sets with unexpected significant overlap, an overlap which deviates from what is expected by chance. Regardless of the test used, the results are often similar but are sensitive to list size [13]. Alternatively, in many cases the ranking of genes/proteins identified experimentally is known. For example, the fold-change or p-value of the observed gene/protein level as it is compared with an experimental control. Hence, several enrichment tools also use non-parametric tests such as Kolmogorov-Smirnov and Wilcoxon tests to compute enrichment with consideration of ranks [5]. For the Lists2Networks system we only implemented the Fisher exact test to compute gene-list enrichment and overlap [12]. The Fisher exact, the binomial proportions, or the Chi-squared tests are based on the null normal distribution assumption which treats gene as independent. Such an assumption is obviously inaccurate but it simplifies the task and capable of producing useful results.

## Implementation and Results

**Figure 1:**
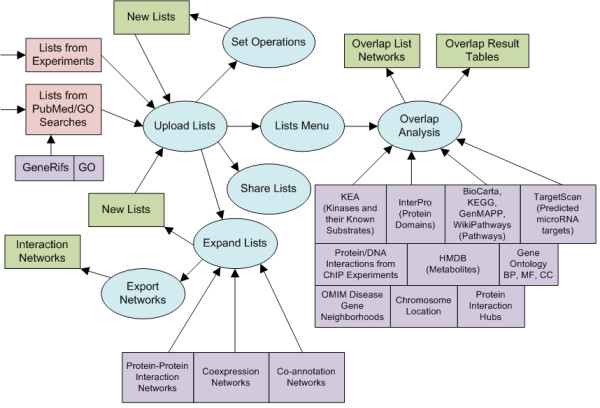
**System architecture of the L2N software**. The system contains several related components (blue ellipses). Registered users are able to upload lists of genes/proteins. Set operations allow users to create new lists from existing lists. Overlap analyses use background databases of gene-list libraries to compute enrichment. Lists of genes are able to be expanded using background knowledge networks of PPIs, co-expression, and co-annotations. Users have the capability to share their lists with other users and communicate with other users through a messaging system.

The Lists2Networks (L2N) system is implemented using a combination of PHP, JSP/Java, and JavaScript. The data is stored on the server side in a MySQL database. To make the application have a look-and-feel of a desktop application Asynchronous JavaScript and XML (AJAX) is used for concurrent updating of parts of the web-site without full page refreshes. The system contains several modules (Fig. 1) which include: an interface to upload lists, an interface to expand lists using background networks, a tool to manipulate lists using set operations, an interface to analyze lists for overlap and enrichment of functional terms, a protein-protein interaction browser, and a user communication system which allows users to share lists with other users. The following is a brief description of the different components/modules of the system:

### The starting page and the user communication system

One of the obvious advantages of a web-based system is the ability to access data from any desired location, not being bound to a single computer. Additionally, data and analysis can be shared in a collaborative way. The start page of L2N provides users with the ability to communicate with other researchers using an integrated messaging system. For this we use a similar approach found on popular social networking sites such as Facebook. The system provides users with the ability to locate other users through a user search utility. Once users identify each-other, a friendship can be initiated by a friend request. After establishing a friendship, users can exchange messages and share gene lists. A message-board displays incoming messages and gene lists sharing requests. By accepting a gene-list, sent by another user, the list is automatically integrated into the user-lists-workspace, ready to be analyzed by the analysis components of the system.

### The upload component

After starting the system for the first time, the user-lists-workspace is empty. In order to populate the workspace, gene lists have to be uploaded to the system. The upload component of the system allows users to upload lists of mammalian genes in Entrez Gene Symbol format. L2N implements four upload options within the upload component of the system: The first option allows users to drag-and-drop multiple text files containing lists of genes into a Java applet. This feature allows fast upload by bypassing the restriction of HTML forms. Alternatively, a standard HTML form can be used. Both the Java Applet and the HTML form allow for annotation of the uploaded gene-list with a detailed text-based description. The third and fourth options for uploading lists of genes are self contained and do not required user data. The third option allows users to enter any search term into a PubMed search. The system uses PubMed e-utilities to return a set of abstracts that match the searched term. These abstracts are converted to a list of human Entrez gene symbols using GeneRifs. GeneRifs is a manually curated dataset that links publications with genes. The resultant gene list can be uploaded into the workspace. The final and forth upload option uses Gene Ontology. Here users can type biological terms in a search box. The matching terms from the Gene Ontology database with the associated genes are then displayed and made ready for upload into the workspace.

### The expand-lists component

**Figure 2:**
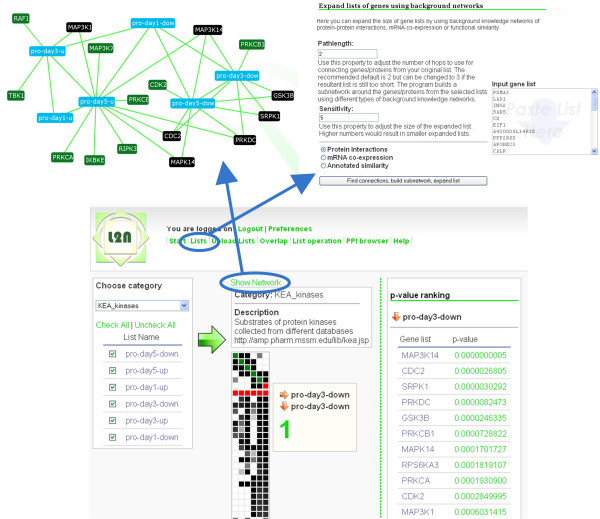
**Bottom center: Screenshot from the overlap component showing sample analysis**. The background knowledge category chosen is KEA kinase-substrate enrichment. Top right: Expand Lists component screenshot. Uploaded lists can be expanded using protein-protein interactions network, co-expression network, or co-annotation network. Users can set two parameters: sensitivity and number of steps. This allows controlling the size of the expanded lists. Top left: Network of lists representation. Uploaded user lists (blue) are connected if they overlap significantly. Green and black nodes represent labeled gene-lists from a specific library (kinases and their known substrates) that overlap with the uploaded lists. Green nodes represent annotated lists that overlap with only one input list whereas black nodes are lists that overlap with two or more input lists.

This component of the L2N system provides users with the ability to expand lists based on networks created from known protein-protein interactions, co-expression correlations, or co-annotation correlations. These background knowledge networks are represented as graphs made of nodes and links [14,15]. Interactions from those networks are used for "connecting" the genes/proteins from input lists similarly to the way we achieved this for the software system Genes2Networks [16]. The shortest paths between pairs of nodes (genes) from the input list are found to form a subnetwork that "connects" the input list nodes using additional genes/nodes from the background network (Fig. 2). The resultant subnetworks are visualized using a Flash-based interactive network viewer that is embedded within the application. Additionally, the output subnetwork, besides being visualized within the web-page, is made available for download in SIF format, amenable for import, analysis and visualization with Cytoscape [17]. Furthermore, the subnetwork that is generated from the input list is automatically converted into a new list that can be added back into the user's workspace as an expanded list. The subnetwork reconstruction process and implementation also contains features that give users the flexibility to set a threshold for inclusion of intermediate nodes and links in the subnetwork. The threshold settings are based on the specificity of the additional proteins/genes (intermediates) to interact with the input list, as well as the number of steps/links used to connect the nodes. The specificity calculation is using the proportions of links to seed nodes from the intermediate nodes compared with total interactions for the intermediate nodes in the background respective network. Intermediates are ranked based on their counts of links in the subnetwork as compared with their total links in the background prior knowledge protein-protein, co-expression or co-annotation networks.

The Expand Lists component gives users the ability to choose the background network to use when expanding lists. There are three options: a protein-protein interactions network, a co-expression network, and a co-annotation network. The protein-protein interaction network is compiled from a variety of experimentally determined mammalian (mouse/rat/human) interactions recorded in the following databases: BioGRID [18], Reactome, Biomolecular Interaction Network Database (BIND) [19], the Human Protein Reference Database (HPRD) [20], IntAct [21], Database of Interaction Proteins (DIP) [22], Molecular INTeractions database (MINT) [23], PDZBase [24], Protein-Protein Interaction Database (PPID) [21,25], as well as the interactions described in references [26-28]. All interactions from these databases/datasets were determined experimentally and include a PubMed reference to the primary source article. For creating the co-expression network we used COXPRESdb [29], a database which contains a downloadable table of co-expressed genes in mouse and human. To create the co-annotation network we defined a pair-wise distance between genes:(1)

The co-annotation is based on the co-appearance in annotated gene-set lists from MSigDB [5]. The pair-wise dependency between genes can be represented as a graph where nodes are genes and edges represent the co-appearance level between two genes in respect to inclusion in an annotated gene-set list.

### The set operation component

Once lists have been uploaded, and if desired expanded, users can apply set operations on lists to generate additional lists. This feature of the system is useful for performing common steps in the analysis of many different experimental data scenarios. For example, it is often desired to obtain a consensus list of genes that appeared in a set of repeated experiments, i.e., genes that appeared to be consistently up-regulated in several microarray experiments. For applying such operation, users can apply the "intersection" function. Similarly, analysis of proteomics data often requires removal of sticky non-specific proteins, for example, removing all ribosomal proteins, which commonly reappear in immuno-precipitation followed by mass-spectrometry (IP-MS) type of experiments. For applying this operation users can apply the "not" function.

### The overlap component

The overlap component of L2N is the most extensive, useful and powerful feature of the system. With this feature users can select certain lists from their library of lists to generate an overlap representation between the loaded lists, as well as overlap with categories of prior biological knowledge in the form of collections of labeled gene-lists stored in the Gene Matrix Transposed (GMT) flat file format [5]. Each GMT file contains rows of gene sets where the first two columns in the file describe the list, while the rest of the entries in each row are Entrez Gene symbols. Similarity among lists is computed using the Fisher exact test and the overlap is visualized as a distance table matrix (Fig. 2). The resultant similarity matrix displays the overlap among user-selected lists and libraries of gene-sets.

The overlap analysis section allows the identification of biological themes that can be associated with multiple lists from the user's-list-workspace. Moreover, lists from the workspace can be studied for similarity to each other and to previously annotated gene-sets from many biological categories (Gene Ontology, Pathways, microRNAs, protein domains, kinases, etc.). A complete list of prior biological knowledge gene-list libraries is provided in Table 1. To start the analysis, a GMT file which represents a category of a biological theme has to be selected. As an example, we show the results when the user chooses the kinase-substrate prior knowledge category (KEA_kinases) applied on a sample set of user inputted lists (Fig. 2). Before the analysis can begin, the user needs to select the gene lists that should be included in the overlap analysis. After selecting the lists and pressing the large green arrow, the overlap matrix is computed and displayed right next to the listings of the gene lists.

**Table 1:** GMT files used for gene-list overlap analysis in L2N

Name	Description	Source	GMT File Creator
WikiPathways_pathways	Biological pathways	http://www.wikipathways.org	Ma'ayan lab
Reactome_pathways	Biological pathways	http://www.reactome.org	Ma'ayan lab
GenMAPP_patwhays	Biological pathways	http://www.genmapp.org	MSigDB
KEGG_pathways	Signaling pathways	http://www.genome.jp/kegg	MSigDB
BioCarta_pathways	Signaling pathways	http://www.biocarta.com	MSigDB
Chromosome_location	Chromosome location	http://genome.ucsc.edu	MSigDB
TFs_predicted_binidng_sites	TRANSFAC predicted sites	http://genome.ucsc.edu	Ma'ayan lab
TFs_chip_interactions	ChIP interactions collected from literature by the Ma'ayan lab	Various sources from individual publications	Ma'ayan lab
Gene_Ontology_BP	Gene Ontology Biological Process	http://www.geneontology.org	Ma'ayan lab
Gene_Ontology_MF	Gene Ontology Molecular Function	http://www.geneontology.org	Ma'ayan lab
Gene_Ontology_CC	Gene Ontology Cellular Component	http://www.geneontology.org	Ma'ayan lab
HMDB_metabolites	Genes associated with Metabolites	http://www.hmdb.ca	Ma'ayan lab
OMIM_disease_genes	Genes associated with a specific disease	http://www.ncbi.nlm.nih.gov/omim	Ma'ayan lab
OMIM_disease_neighborhoods	Genes associated with a specific disease expanded using protein interactions and the genes2networks algorithm	http://www.ncbi.nlm.nih.gov/omim and http://actin.pharm.mssm.edu/genes2networks	Ma'ayan lab
KEA_kinases	Substrates of protein kinases collected from different databases	http://amp.pharm.mssm.edu/lib/kea.jsp	Ma'ayan lab
PFAM_InterPro_domains	Proteins that share a structural domain	http://pfam.sanger.ac.uk and http://www.ebi.ac.uk/interpro	Ma'ayan lab
Predcited_microRNAs	Predicted microRNAs that bind to mRNA of sets of genes	http://www.targetscan.orghttp://microrna.sanger.ac.uk/sequences	Ma'ayan lab
Protein_interaction_hubs	Proteins that interacts with many other proteins	Protein interactions collected from several mammalian protein interactions databases http://actin.pharm.mssm.edu/genes2networks	Ma'ayan lab

Columns include: a) File name as it appears in the drop-down box in the overlap feature on the web-page; b) Description of the content of the gene-list library; c) URL of the source of the data. d) Library created by.

The screenshot in Fig. 2 shows the results after a sample overlap matrix was created. The matrix itself is interactive. By hovering over the squares in the matrix, information about the content of each square is displayed on the right panel. The large green numbers in Fig. 2 are the p-values of the overlap enrichment computation using the Fisher exact test before applying the Bonferroni or Benjamini-Hochberg corrections. By clicking on the red squares from the row of red squares, columns are sorted by their p-values which represent overlap with annotated gene-list libraries. After the sorting is done a table of ranked enriched terms is displayed on the right panel. The row of red squares, used for sorting, separates the matrix into two separate sections. Below the red line is the overlap between the input gene lists and the lists belonging to the chosen biological category. Each column of the matrix represents one of the input files, whereas each row below the red line represents a labeled gene list from a prior knowledge library. It is also possible to click on any of the squares of the matrix to see the genes that overlap. The matrix allows fast browsing of enriched functional annotations that match many input lists. Furthermore, the enrichment of terms from different categories associated with many input lists can be compared easily where common biological themes can be identified.

In addition to the overlap matrix display, users can view overlap between input lists as a network. Such network can be displayed by clicking the "Show Network" button on top of the overlap matrix (Fig. 2). This network visualization displays the input files as nodes, as well as enriched gene lists from a specific prior knowledge category as nodes. Only gene lists that are enriched (having high overlap) with at least one other list after the Bonferroni correction with a p-value < 0.05 are connected with an edge and included in the network for visualization. An edge (link) in the network represents a significant overlap between pairs of lists. Input lists are colored in blue, enriched gene lists from a prior knowledge category are in green or black. Black nodes are gene lists from the prior knowledge category that have significant overlap with more than one input list. In the example in Fig. 2 the biological category is KEA_kinases and it shows that different lists of proteins from the input lists are associated with different kinases.

The data for the prior biological knowledge enrichment analyses was created using original GMT files we developed, as well as few GMT files downloaded from MSigDB [5] (Table 1). The original GMT files (gene-list libraries) that we created are: pathways from WikiPathways, data from ChIP experiments, predicted microRNA-mRNA interactions from miRBase and TragetScan, kinase-substrate interactions from KEA, protein-metabolite interactions from HMDB, disease genes from OMIM, disease-gene neighborhoods using OMIM and Genes2Networks, protein interaction hubs using Genes2Networks and protein structural domains using PFAM and InterPro. Additional available libraries previously created by others are: pathways from KEGG, BioCarta and GenMAPP, as well as chromosomal location [30]. To generate the microRNAs GMT file we processed the data from miRBase [31] and TargetScan databases. Such databases contain gene lists predicted to be regulated by microRNA families. For the kinases, we used a database of experimentally determined kinase-substrate interactions we recently developed for KEA [32] by consolidating several web-based resources reporting kinase-substrate relations. The metabolites GMT file was created from data downloaded from HMDB [33], and the disease neighborhood GMT file was created from lists of genes from OMIM [34] and expanded using protein-protein interactions as described above. Expanding lists of disease genes using known protein-protein interactions assisted us in discovering SHOC2 as a novel Noonan Syndrome disease causing gene [35] justifying the disease gene neighborhood concept. The gene-lists libraries will be updated manually periodically. Specifically we are mostly interested in updating the protein-protein interactions data, kinase-substrate interactions data, datasets from RNAi and ChIP screening, and microRNA-mRNA target interactions. Such datasets will be quality controlled using manual and automated filtering methods. Users are welcome to contribute gene-list libraries to the system. However, these contributions will be monitored by the authors for quality.

### The list sharing component

Since the system is web-based, we provide users with the ability to share lists and communicate results and messages with other users through a dedicated messaging system. The system provides users with the ability to locate other users through a user search utility. Once users identify each other and want to communicate and share lists with one another, a friendship request message can be initiated. Such request needs to be approved by the requested party for establishing communications. Once such friendship has been established, both users can share lists and exchange messages.

### The protein-protein interactions browser component

Additional feature that is desired by experimental and computational biologists is to explore which proteins directly or indirectly interact with a specific protein of interest. It is also desired to see how lists of interactors of one protein overlap with other experimentally developed lists. For example, results from IP-MS proteomics experiments, pulling down and characterizing interactions for specific protein baits are logically compared to already known interactions for specific proteins based on literature and other resources that previously characterized protein-protein interactions. This can be used to assess how consistent the IP-MS results are with what is already known about protein-protein interactions with the bait. For this, L2N has a protein-protein interactions browser feature where users can quickly identify all direct interactors for a specific gene/protein. Users can upload lists of interactors as input lists for comparison, enrichment, expansion, and visualization, as part of the integrated analysis provided by the other parts of the L2N system. The implementation of such browser is delivered as a dynamical text-based expansion system where the original gene/protein is selected from a list and then the lists of direct interactors are dynamically displayed in a recursive manner. Protein-protein interactions have been compiled as described above.

### Flash based network viewer

To visualize networks within a web-page in a dynamic representation, we used Flash/ActionScript3 which allows the efficient development of interactive web content. The advantage of using Flash over other recent web technologies such as JAVA applets and AJAX is that Flash/ActionScript3 integrates the classes of Sprites, which are a powerful vector graphics entity with attached action listeners for user interaction. Since the latest version of ActionScript (AS3), the programming language used in Flash is no longer restricted as with previous versions. AS3 has strong emphasis on visual output and user interactivity, making it ideal for dynamic web-based network visualization purposes. The network viewer is implemented using a force directed layout algorithm to place nodes by minimizing a stress function considering optimal edge length and node repulsion.

### Case study: integrating proteomics and phosphoproteomics studies applied to profile embryonic stem cell differentiation

To illustrate how L2N can be utilized to integrate results from different but related high-content genome-wide profiling studies, we created a case study (Additional file 1). We integrated and analyzed data from the following four proteomics and phosphoproteomics studies applied to profile differentiating mouse and human embryonic stem cells: Lu et al. [36] who profiled the nuclear proteome after silencing of Nanog; two phosphoproteomics studies of human embryonic stem cells driven to differentiate by two different methods [37,38]; and the Nanog interactome as determined by a serial set of proteomics experiments [39]. Although our focus and aim of the case study is to demonstrate to novice users the capabilities of the L2N software system, we obtained some interesting results. For example, there are 23 proteins that overlap between the Nanog-KO-Nuclear-Day5-Up from the Lu et al. study and the Brill et al. list of phosphoproteins identified four days after inducing differentiation with retinoic acid. This is a statistically significant overlap with a p-value of ~0.000002 (Fisher exact test). The proteins from this list are great candidates for further functional experimental validation and characterization as components of an early differentiation pathway. Additionally, to further identify proteins that potentially belong to the Nanog interactome, we cross referenced an expanded subnetwork made of the Nanog interactome reported by Wang et al. and the expand list feature of L2N with the Lu et al.-Day5-Down-List. We found that EED, JARID1B, PNO1, SMARCA5 and UTF1 are identified in both lists from such cross-reference analysis. These candidates should be further validated as bona-fide self-renewal components belonging to the Nanog interactome. EED and JARID1B are already known components of the self-renewal machinery as was discovered recently (more details can be found in the Case Study provided with this manuscript as Additional file 1).

## Conclusions

Lists2Networks is a user friendly powerful system that is expected to significantly ease the computational analysis process of experimental biologists employing high-throughput experiments at multiple layers of regulation. It simplifies gene-list enrichment analyses and provides means to expand lists with three types of prior biological knowledge networks, apply list operations, browse protein-protein interactions and communicate lists between users. The ability to form networks of lists based on list similarity is a powerful method to integrate many different experiments with background knowledge for hypothesis generation. While other similar tools may already exist, L2N has several features not implemented in existing systems or partially implemented in other systems. These include, for example, the ability to expand lists using protein-protein interactions, co-expression data, and co-annotation data; the ability to share lists and communicate by a messaging system in a collaborative fashion; web-based application of list operations such as union, complementation and subtraction; never before assembled prior knowledge gene-lists libraries such as: data collected from high-throughput ChIP experiments, expanded disease gene networks, protein interaction hubs, and kinase substrates; uploaded lists are saved on the server not requiring users to re-upload lists every time they used the system whereas users can manually edit the content of lists with built-in synonym resolution feature. Additionally, users can create original gene lists using any search term through PubMed and GeneRifs or easily integrate lists from the Gene Ontology database.

In regards to future plans, we can implement the system for other model organisms and improve the ID matching features. Additionally, we plan to provide plug-ins that would enable the exporting and importing of lists using other software systems. In summary, L2N stands out being user-friendly, web-based, robust and simple, making it more accessible for novice users as compared with other existing academic or commercial packages aim to address similar needs.

## Availability and Requirements

Project name: Lists2Networks

Project home page: http://www.lists2networks.org

Programming languages: PHP, Java, MySQL, ActionScript/Flash

Requirement: Web browser and internet access, Java support, JavaScript enabled browser, Flash Player 10

## Authors' contributions

AL and AM designed the system. AL implemented the system. AM wrote the manuscript.

## Supplementary Material

Additional file 1 **Lists2Networks Case Study: Integration of Proteomics Data Applied to Embryonic Stem Cells**. A demonstration of how to use Lists2Networks for the analysis of protein lists collected from several proteomics and phosphoproteomics studies that profiled differentiating mouse and human embryonic stem cells. Click here for file
