# Supplementary material for: Lists2Networks: Integrated analysis of gene/protein lists
Source: BMC Bioinformatics. 2010 Feb 12;11:87. doi: 10.1186/1471-2105-11-87 (PMC2843617; doi:10.1186/1471-2105-11-87)
Supplement: Additional file 1 — Lists2Networks Case Study: Integration of Proteomics Data Applied to Embryonic Stem Cells. A demonstration of how to use Lists2Networks for the analysis of protein lists collected from several proteomics and phosphoproteomics studies that profiled differentiating mouse and human embryonic stem cells. [file 1471-2105-11-87-S1.PDF]

Lists2Networks

Case Study

Alexander Lachmann and Avi Ma'ayan

Ma'ayan Lab

January 5st, 2010

MOUNT SINAI SCHOOL OF MEDICINE

Integration of Proteomics Data Applied to Embryonic Stem Cells

Motivation

Understanding the molecular regulatory circuitry of embryonic stem cells has recently been the primary focus of many research laboratories. This surge of interest has been fueled by the discovery that somatic cells can be converted into cells that resemble embryonic stem cells, namely Induced Pluripotency Stem Cells (iPS cells). Since embryonic stem cells can be maintained indefinitely in culture and can be driven to differentiate into all types of somatic cells, the finding that such cells can be created from somatic cells removes ethical barriers and tissue rejection issues for regenerative medicine applications. Stem cells researchers have been utilizing systems biology strategies to dissect the regulatory networks governing self-renewal and differentiation by applying genome-wide profiling methods. Due to limitations in available biotechnologies that can be used to profile proteins at a genome-wide level, until recently, most genome-wide studies of stem-cells have been measuring protein-DNA interaction, mRNA and microRNA, and chromatin structure and methylation status. However, recently several studies published results of applying mass-spectrometry proteomics to profile embryonic stem cells. Such studies can provide direct assessment of the cell signaling networks controlling embryonic stem cells self-renewal and differentiation. The cell signaling pathways in stem cells are much less understood as compared with the gene regulatory networks and chromatin modification changes. In this case study we demonstrate how Lists2Networks can be used to integrate results from four proteomics studies applied to embryonic stem cells to form novel hypotheses that can be tested experimentally, as well as shed-light on some cell signaling regulatory mechanisms that govern embryonic stem cell differentiation. Specifically, we use Lists2Networks to integrate and analyze data from the following four proteomics studies: Lu et al. [1] who profiled the nuclear proteome after silencing of Nanog; two phosphoproteomics studies of human embryonic stem cells driven to differentiate by two different methods [2, 3]; and the Nanog interactome as determined by serial set of AP-MS experiments [4]. Although the results from our analyses are interesting, our focus and aim here is to demonstrate to novice users the capabilities of the Lists2Networks software system.

Creating the project workspace

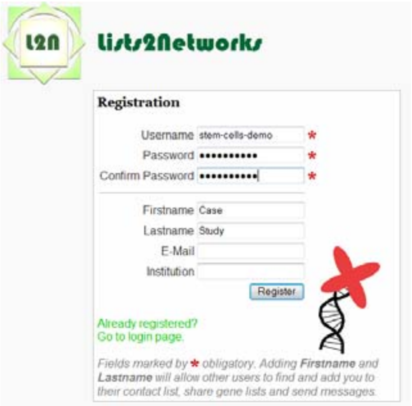

To start a project, users need to create an account. For this project we registered an account with the user name "stem-cells-demo". The e-mail and affiliation information are not required. However, we did input a user name for being able to exchange lists with other users. We set the First Name to "Case" and last name to "Study".

Go to: *http://amp.pharm.mssm.edu/lachmann/upload/register.php*

Enter a username and password to create an account.

Once the account was created, we are automatically signed in and can see the following page:

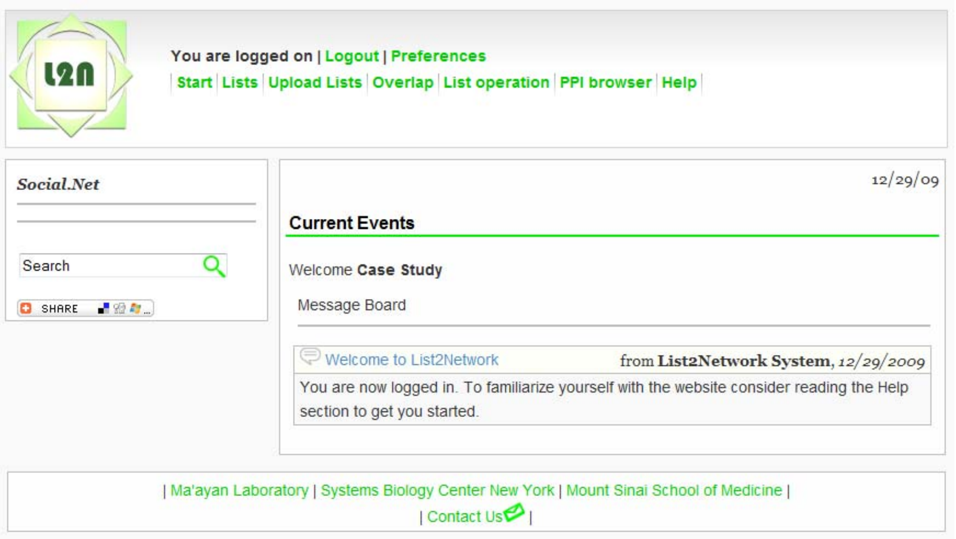

Constructions of lists to compare

The first step for the analysis of the embryonic stem cell proteomics datasets is to convert and construct lists of genes from the proteomics studies for upload onto Lists2Networks. For this we need to visit the publications mentioned above and extract the lists from such studies. Since we know that the lists from Lu et al. [1], the Nanog knock-down followed by the nuclear MS proteomics, has been already extracted by the user Avi Maayan, we will ask Avi to share these lists with us instead of constructing such lists from scratch. For this we will first send Avi a friend request. For this we will use the search function to find Avi:

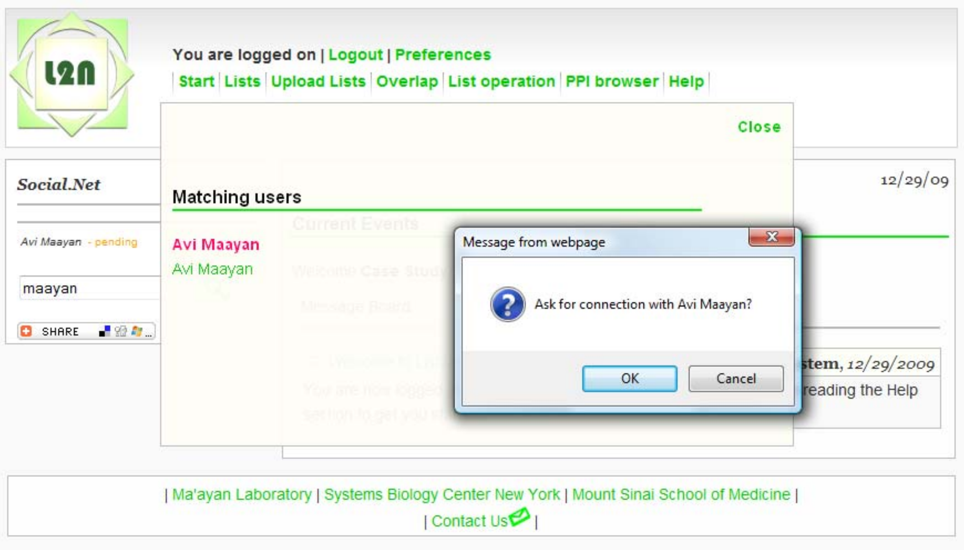

Clicking on Avi's name, we can request to be his friend. Our friendship status with Avi will become pending. After Avi accepts our friendship, he will share the proteomics lists from the Nanog knock-down study with us. Once he has done this our "Start" screen will look as follows:

Go to:
http://amp.pharm.mssm.edu/lachmann/upload/help.php

Download "Case Study Files".

Go to:
http://amp.pharm.mssm.edu/lachmann/upload/upload.php

Unzip and drag files into the "Upload Applet" and press "Upload".

Alternatively use the HTML form to upload each file separately

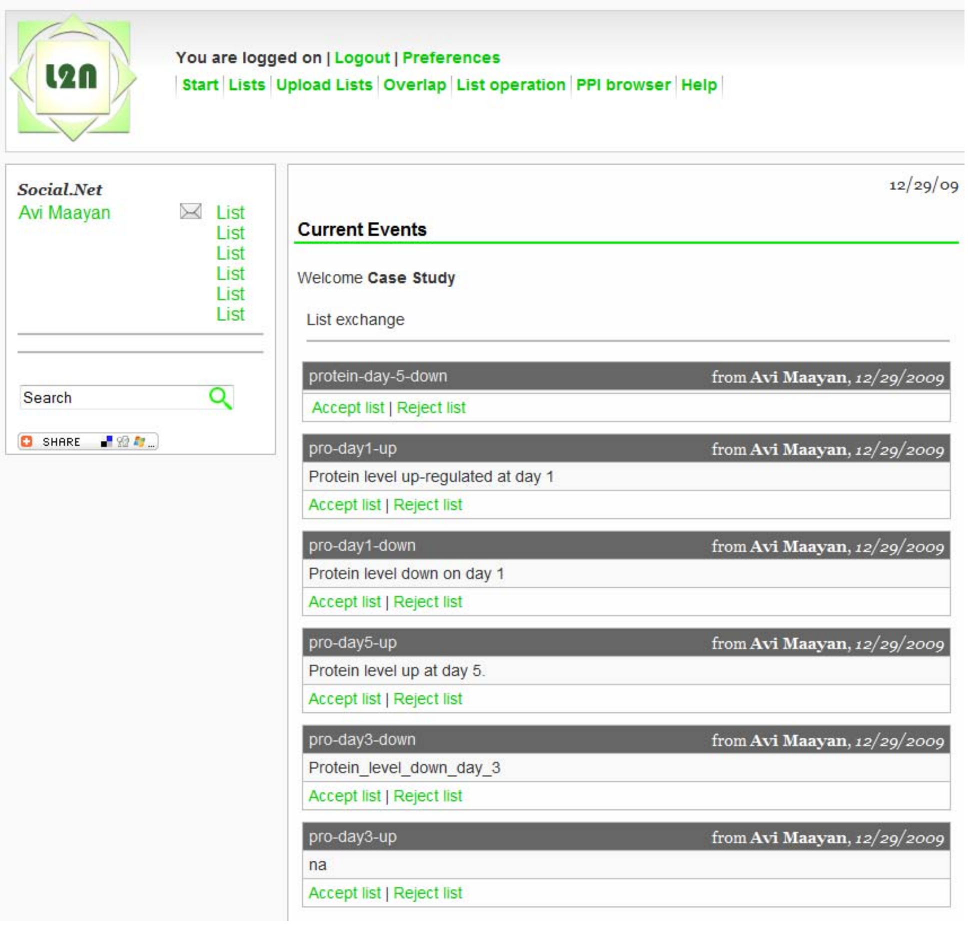

We will now "Accept" all the lists from Avi and then click on the Lists tab to view the lists' content.

Since we plan to compare these lists to other lists, we will rename the six lists using a more descriptive name that indicates that these lists are from a Nanog

knock-down time course nuclear proteomics study. To do this we need to click on the pencil icon next to each list.

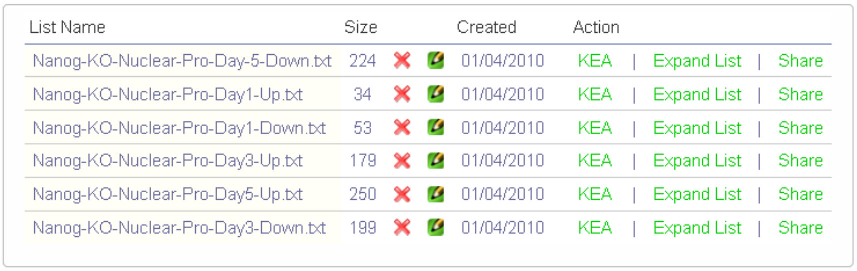

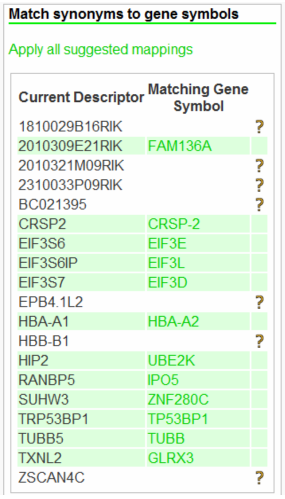

At the same time we will also let the system map automatically gene IDs. An example screen for such matches is shown on the left.

We are now ready to upload lists from the phosphoproteomics studies. The Brill et al. Cell Stem Cell 2009 study [2] reported proteins that are increasingly phosphorylated in undifferentiated and differentiated hES cells (four days after RA stimulation). The lists of proteins are reported in the supplementary tables 3A and 4A of the paper. We can cut and paste the gene symbols from the Excel files and upload the lists onto Lists2Networks using the manual upload option as seen below:

In this example we ignore information about phosphosites, the quantity of the measurements, and note that the study was applied to human ES cells whereas the Nanog-KO proteomics data was collected for mouse. Regardless, we will show how Lists2Networks can help us to quickly extract interesting insights from merging these different proteomics datasets.

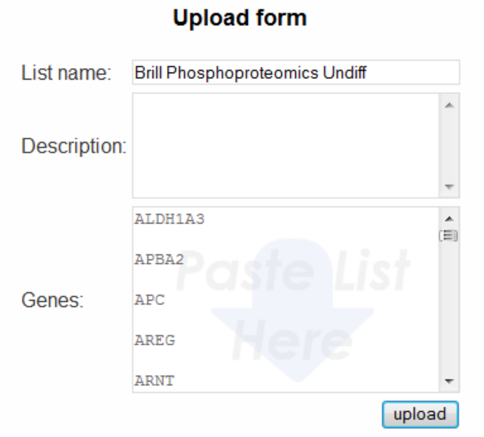

The second phosphoproteomics study by Van Hoof et al. [3] also profiled human ES cells. Similarly to the Brill et al. study the results from this study are reported in Excel files as supporting online materials. We extracted the results from Table S3 by setting up arbitrary cutoffs. We only took the lists from the 4 hour time point for this case study. In contrast with the Brill et al. study, Van Hoof et al. measured the changes in phosphorylation right after the stimuli as a time-course (30 minutes, 60 minutes and 4 hours after stimulation). Their method to differentiate the cells was also different. They treated the hES cells with BMP and removed growth factors from the media.

The last stem-cells proteomics study that we integrated is from Wang et al. [4]. The uploaded list is called Wang-Nanog-Interactome.

List Name	Size	Created	Action
Wang-Nanog-Interactome.txt	36	01/04/2010	KEA | Expand List | Share
Van_Hoof_Phosphoproteomics_4hr_Diff_Down.txt	186	01/04/2010	KEA | Expand List | Share
Van_Hoof_Phosphoproteomics_4hr_Diff_Up.txt	229	01/04/2010	KEA | Expand List | Share
Brill_Phosphoproteomics_Diff.txt	516	01/04/2010	KEA | Expand List | Share
Brill_Phosphoproteomics_Undiff.txt	364	01/04/2010	KEA | Expand List | Share
Nanog-KO-Nuclear-Pro-Day1-Down.txt	53	01/04/2010	KEA | Expand List | Share
Nanog-KO-Nuclear-Pro-Day1-Up.txt	34	01/04/2010	KEA | Expand List | Share
Nanog-KO-Nuclear-Pro-Day-5-Down.txt	224	01/04/2010	KEA | Expand List | Share
Nanog-KO-Nuclear-Pro-Day3-Up.txt	179	01/04/2010	KEA | Expand List | Share
Nanog-KO-Nuclear-Pro-Day3-Down.txt	199	01/04/2010	KEA | Expand List | Share
Nanog-KO-Nuclear-Pro-Day5-Up.txt	250	01/04/2010	KEA | Expand List | Share

Expanding the Nanog interactome

Since the Nanog interactome list is fairly short, with a length of only 36 proteins, we would like to expand the list by additional related proteins. To do this we can use Lists2Networks by selecting the "Expand List" option displayed as a link right next to the uploaded list name. This will bring up the screen shown below:

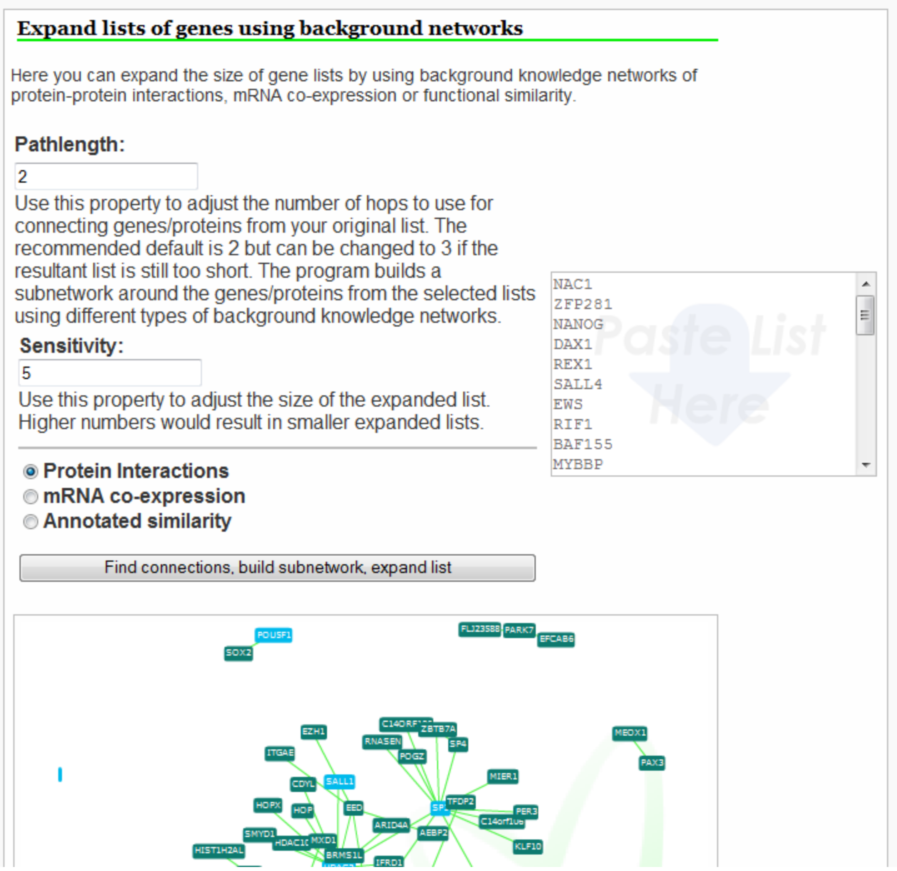

Go to:
http://amp.pharm.mssm.edu/lachmann/upload/viewlists.php

Press "Expand List" next to "Wang Nanog Interactome".

Leave the settings with the defaults and press "Find connections". After the results are returned, change the list name to "Nanog Interactome Expanded" and press "Upload"

Keeping the default settings, using known protein interactions, the list is expanded by 186 intermediates. We then changed the name of the resultant expanded list and press the Upload button to add the expanded list to our library of lists:

List Name	Size			Created	Action		
nanog-interactome-expanded	222	✕	✎	01/04/2010	KEA	Expand List	Share
Wang-Nanog-Interactome.txt	36	✕	✎	01/04/2010	KEA	Expand List	Share
Van_Hoof_Phosphoproteomics_4hr_Diff_Down.txt	186	✕	✎	01/04/2010	KEA	Expand List	Share
Van_Hoof_Phosphoproteomics_4hr_Diff_Up.txt	229	✕	✎	01/04/2010	KEA	Expand List	Share
Brill_Phosphoproteomics_Diff.txt	516	✕	✎	01/04/2010	KEA	Expand List	Share
Brill_Phosphoproteomics_Undiff.txt	364	✕	✎	01/04/2010	KEA	Expand List	Share
Nanog-KO-Nuclear-Pro-Day1-Down.txt	53	✕	✎	01/04/2010	KEA	Expand List	Share
Nanog-KO-Nuclear-Pro-Day1-Up.txt	34	✕	✎	01/04/2010	KEA	Expand List	Share
Nanog-KO-Nuclear-Pro-Day-5-Down.txt	224	✕	✎	01/04/2010	KEA	Expand List	Share
Nanog-KO-Nuclear-Pro-Day3-Up.txt	179	✕	✎	01/04/2010	KEA	Expand List	Share
Nanog-KO-Nuclear-Pro-Day3-Down.txt	199	✕	✎	01/04/2010	KEA	Expand List	Share
Nanog-KO-Nuclear-Pro-Day5-Up.txt	250	✕	✎	01/04/2010	KEA	Expand List	Share

Adding literature-based lists using Gene-Rifs and Gene Ontology

Finally, we wish also compare the proteomics lists to known stem cell biology. For this we can use Lists2Networks' PubMed and Gene Ontology genes-list search. These options are provided when clicking on the Upload tab. To create a canonical embryonic stem cell list we type the search terms

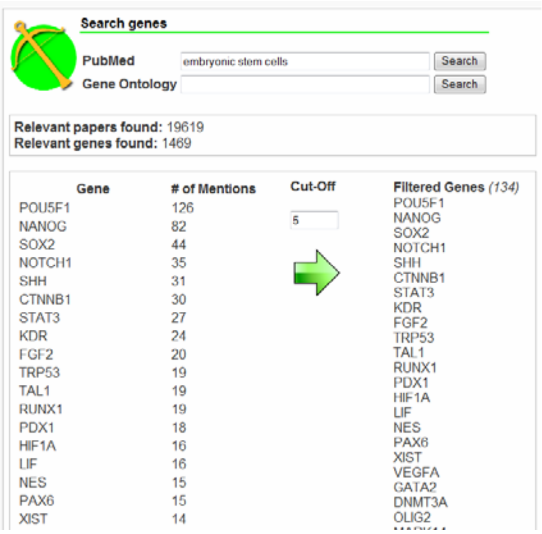

"embryonic stem cells" in the PubMed search textbox and press the search button. The search uses PubMed e-utilities and GeneRifs to compose a list of genes that can be filtered based on the number of occurrences. We filter the list using the default settings, which is 5 occurrences, and then upload the resultant list into our list workspace.

Similarly, we use the Gene Ontology search to add lists that are associated with a Gene Ontology term. We first type in the search box "stem cell" to get all GO terms that contain "stem cell". We then pick "GO:0019827 stem cell maintenance" and "GO:0048863 Stem Cell Differentiation" then upload these lists one by one to our final set of lists ready for the overlap analysis.

Final set of lists we generated are shown below:

Go to:
http://amp.pharm.mssm.edu/lachmann/upload/upload.php

Enter in the PubMed text box: "embryonic stem cells" and use cut-off 5, then upload the gene names that are automatically have been populated the upload form.

Enter in Gene Ontology text box: "stem cell". Then click on the orange arrow next to "GO:0019827" and "GO:0048863" to populate the upload form.

List Name	Size	Created	Action
GO:0048863 Stem Cell Differentiation	35	01/04/2010	KEA | Expand List | Share
GO:0019827 Stem Cell Maintenance	26	01/04/2010	KEA | Expand List | Share
embryonic stem cells in PubMed	135	01/04/2010	KEA | Expand List | Share
nanog-interactome-expanded	222	01/04/2010	KEA | Expand List | Share
Wang-Nanog-Interactome.txt	36	01/04/2010	KEA | Expand List | Share
Van_Hoof_Phosphoproteomics_4hr_Diff_Down.txt	186	01/04/2010	KEA | Expand List | Share
Van_Hoof_Phosphoproteomics_4hr_Diff_Up.txt	229	01/04/2010	KEA | Expand List | Share
Brill_Phosphoproteomics_Diff.txt	516	01/04/2010	KEA | Expand List | Share
Brill_Phosphoproteomics_Undiff.txt	364	01/04/2010	KEA | Expand List | Share
Nanog-KO-Nuclear-Pro-Day1-Down.txt	53	01/04/2010	KEA | Expand List | Share
Nanog-KO-Nuclear-Pro-Day1-Up.txt	34	01/04/2010	KEA | Expand List | Share
Nanog-KO-Nuclear-Pro-Day-5-Down.txt	224	01/04/2010	KEA | Expand List | Share
Nanog-KO-Nuclear-Pro-Day3-Up.txt	179	01/04/2010	KEA | Expand List | Share
Nanog-KO-Nuclear-Pro-Day3-Down.txt	199	01/04/2010	KEA | Expand List | Share
Nanog-KO-Nuclear-Pro-Day5-Up.txt	250	01/04/2010	KEA | Expand List | Share

To change the names of the uploaded lists use the edit function (pencil icon).

Overlap analysis

Now that we have gathered all the needed lists, we can perform the overlap analysis which is the main feature of the Lists2Networks software. The first step will be to run the overlap analysis with the first default prior biological knowledge dataset that is BioCarta pathways. For this we first select the Overlap tab, then "Check All" lists and then run the program by clicking on the green arrow. The results are displayed in a form of a matrix as shown below:

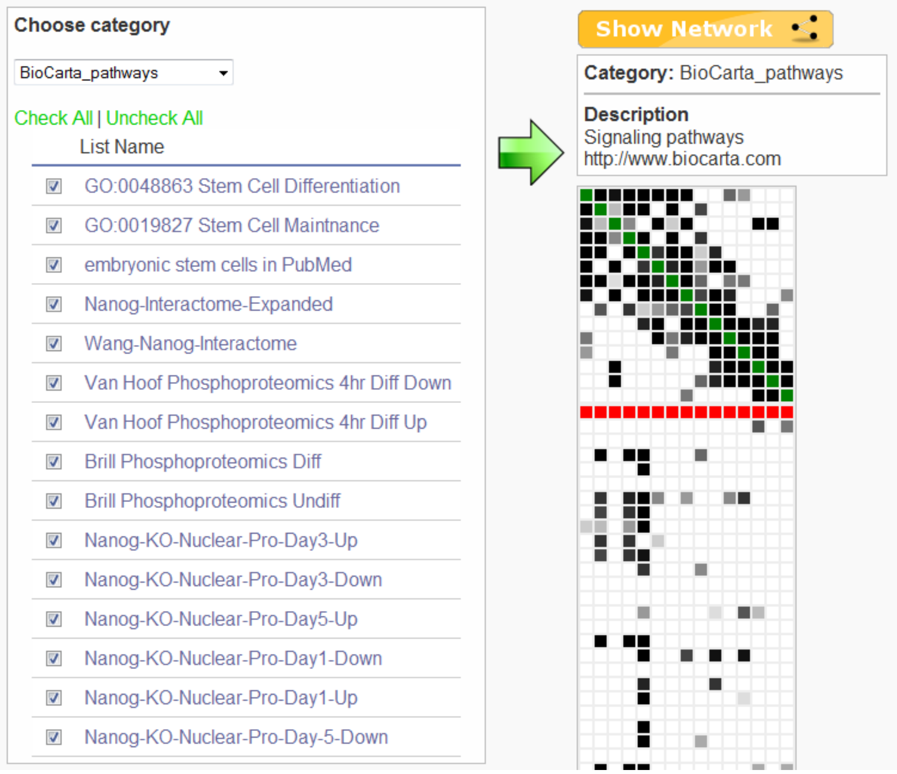

Go to:
http://amp.pharm.mssm.edu/lachmann/upload/overlapmatrix.php

Press "Check All" and press on the green arrow.

(The matrix might not look exactly the same as the clustering algorithm is not deterministic)

We are first interested to see how our input lists overlap and cluster with one another. We noticed that the program automatically detected few clusters. This can be seen by regions of black squares close to the diagonal. Black squares

signify high overlap. Let us focus and examine the cluster of lists at the bottom right of the matrix:

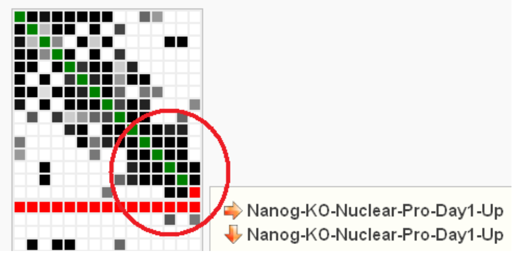

The lists in this cluster include Nanog-KO-Nuclear-Day1-Up, Nanog-KO-Nuclear-Day3-Up, Nanog-KO-Nuclear-Day5-Up, Brill-Phosphoproteomics-Diff, Van Hoof Phosphoproteomics 4hr Diff Down and Diff Up. This is interesting since it shows that the nuclear proteome “up proteins” from the Nanog knock-down study significantly overlaps with the proteins detected to be phosphorylated in the Brill and Van Hoof studies after differentiation. Hence, as the ES cells differentiated, regardless of the experimental conditions or the organism, some proteins increased in their level of phosphorylation as well as increased in overall level in the nucleus. These proteins are likely to be important components of pathways that are turned on once the ES cells begin to differentiate. To get a list of such proteins we can simply click on any desired black square.

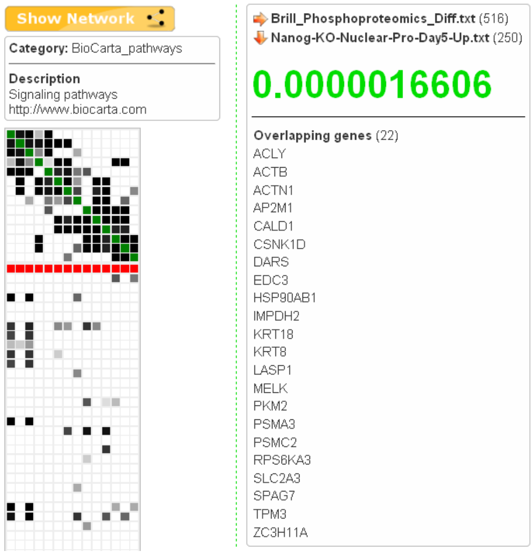

For example, there are 22 proteins that overlap between the Nanog-KO-Nuclear-Day5-Up and the Brill-Phosphoproteomics-Diff list. This is statistically significant with a p-value ~0.000002 (Fisher exact test). The proteins from this list are great candidates for further functional experimental validation and characterization.

The cluster of the proteins that are up from the Nanog knock-down study and the phosphorproteomics can be also be seen when we visualizing such overlap as a network of lists. We can do that by simply clicking on the Show Network button:

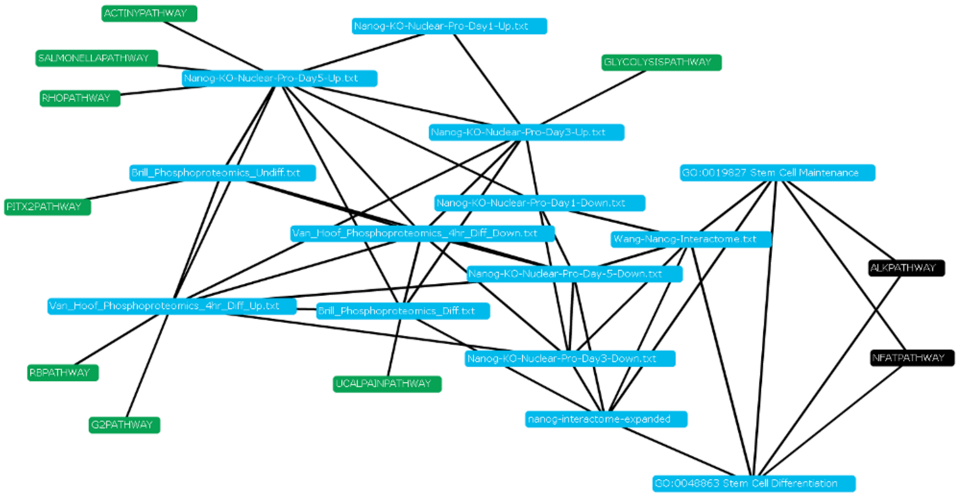

To view the network of lists, press the show network button.

In this network display all the lists from the experiments are shown as blue nodes. The nodes are connected if there is a significant overlap (after Bonferroni correction) between pairs of lists. The input lists, shown in blue are also linked to pathways from BioCarta if they overlap with genes-lists from the BioCarta pathway database.

Similar analysis can be performed with other prior knowledge gene-list libraries. We use the kinase-substrate library to detect the protein kinases that are likely becoming activated or inhibited during differentiation or self-renewal states. For this we will only select the lists from the phosphoproteomics studies and change the category to KEA_kinases as shown below:

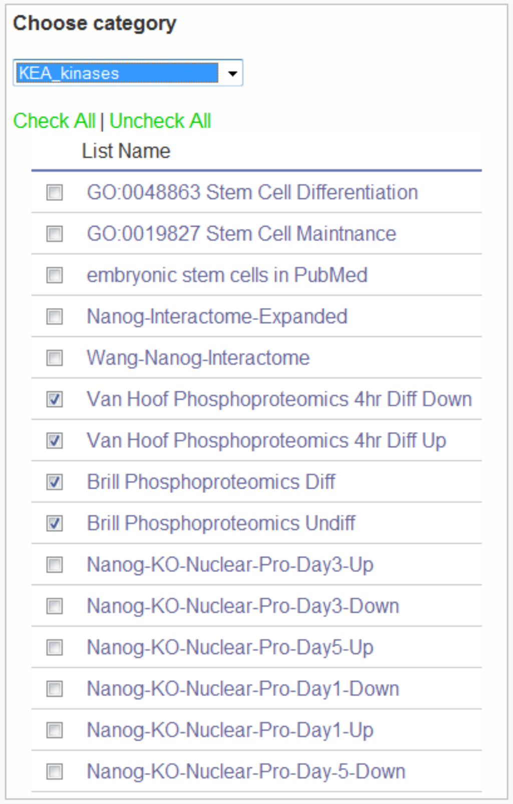

The black nodes are kinases that are connected to more than one input phosphoproteomics list. Further examination of the links in this network show that the Van Hoof lists, although contain similar proteins as compared with the Brill lists, are less selective. This does not mean that their experiments are of lesser value or quality but that these lists are not as useful for separating kinases that are important during undifferentiated vs. differentiated states if we do not consider quantitative measures. Also, let us remind that Van Hoof et al. measured changes after 4 hours, whereas Brill et al. measured changes after 4 days. Additionally, our method of extracting the lists from the results reported by the Van Hoof et al. study may need improvement.

Go to:
http://amp.pharm.mssm.edu/lachmann/upload/overlapmatrix.php

Choose category:
KEA_kinases

Select:
Van Hoof Phosphoproteomics 4hr Diff Down
Van Hoof Phosphoproteomics 4hr Diff Up
Brill Phosphoproteomics Diff
Brill Phosphoproteomics Undiff

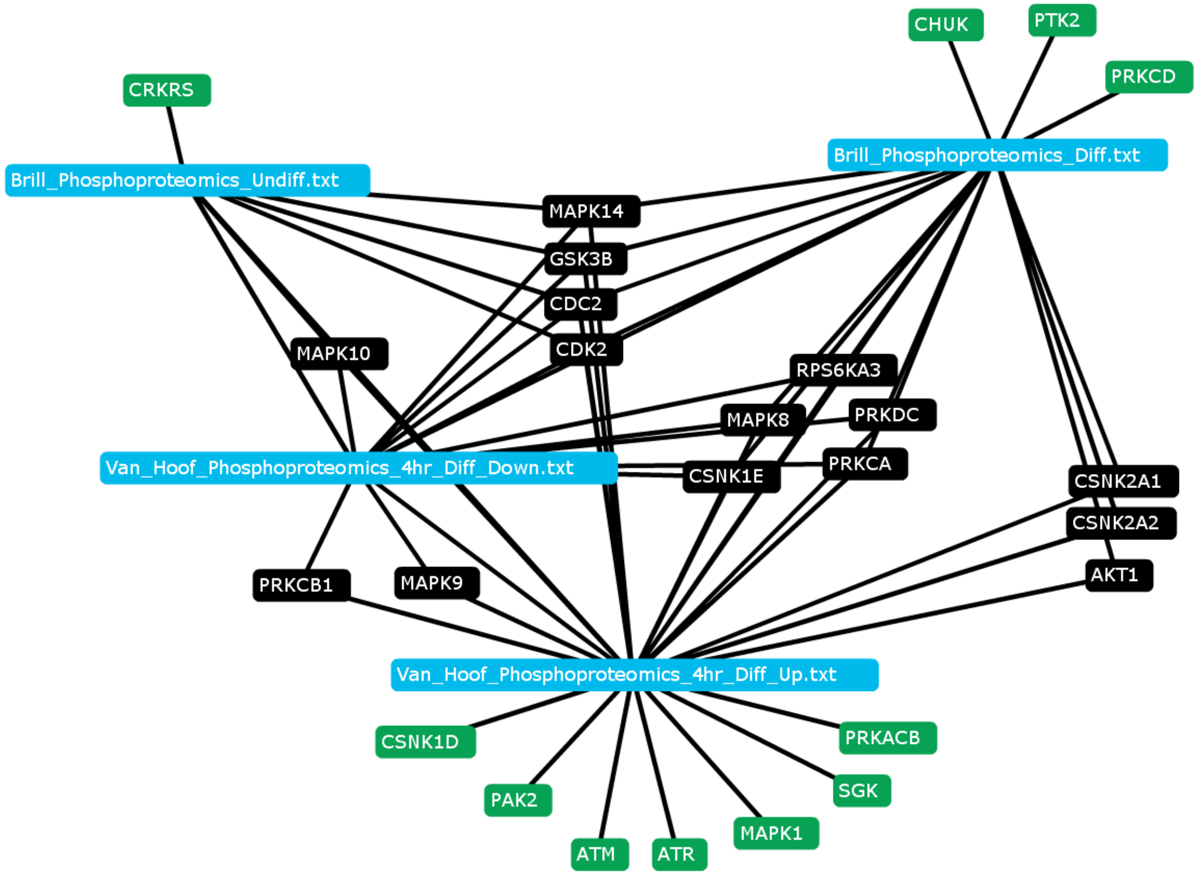

If we only look at the kinases enriched for the lists from the Brill et al. study the results are clearer, showing the kinases that are potentially active when the cells are differentiated or undifferentiated. Four kinases are enriched for both lists: CDC2, GSK3B, MAPK14 and CDK2.

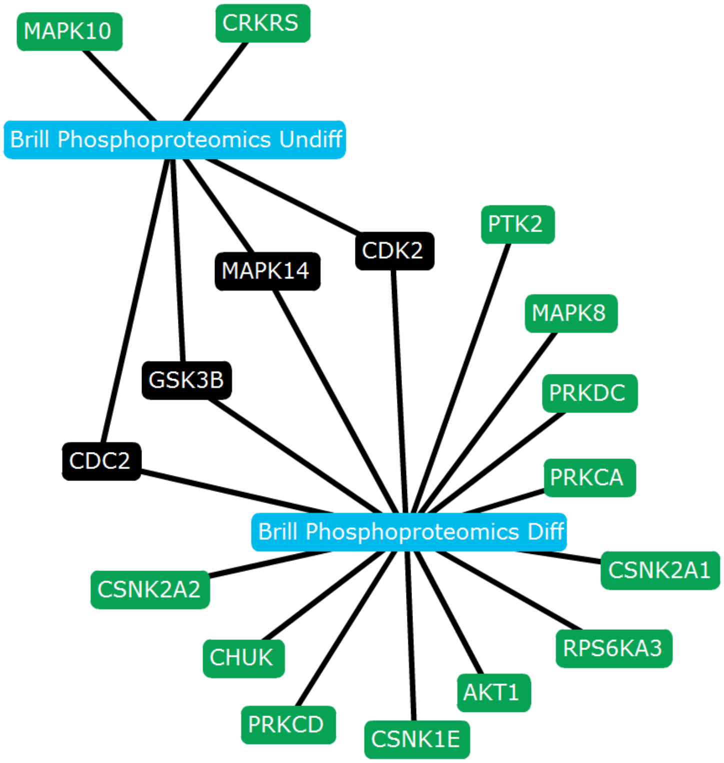

Go to:
http://amp.pharm.mssm.edu/lachmann/upload/overlapmatrix.php

Choose category:
KEA_kinases

Select:
Brill Phosphoproteomics Diff
Brill Phosphoproteomics Undiff

These results can also be provided in a tabular format by clicking the red squares in the matrix as shown below:

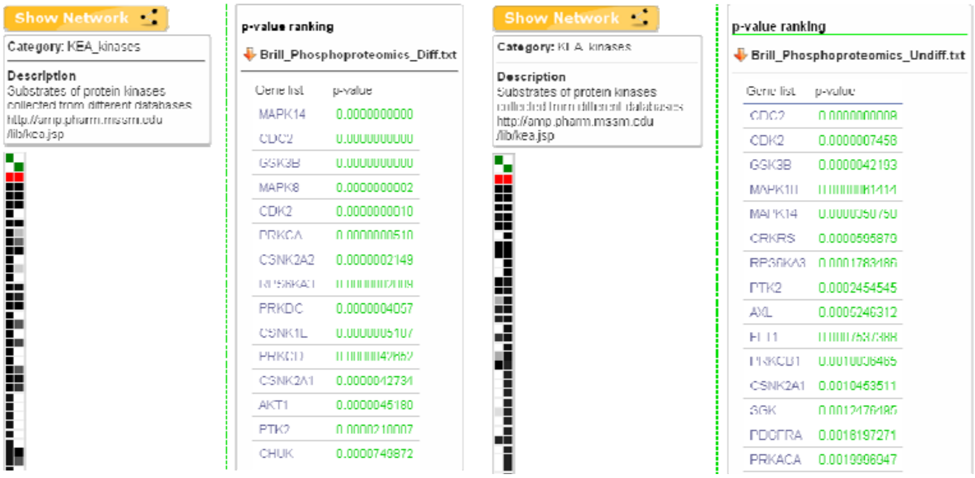

In summary, this analysis links changes in phosphorylation with the protein kinases that are likely responsible for the observed changes. The kinases listed at the top are great candidates for further functional examination. The substrates of these kinases are available for examination by clicking on the black squares.

So far our focus was on the networks active during early differentiation, we would now like to begin to examine the self-renewal network. This time we will select the proteins that went down in the Nanog-knock-down study and the lists from the Nanog interactome:

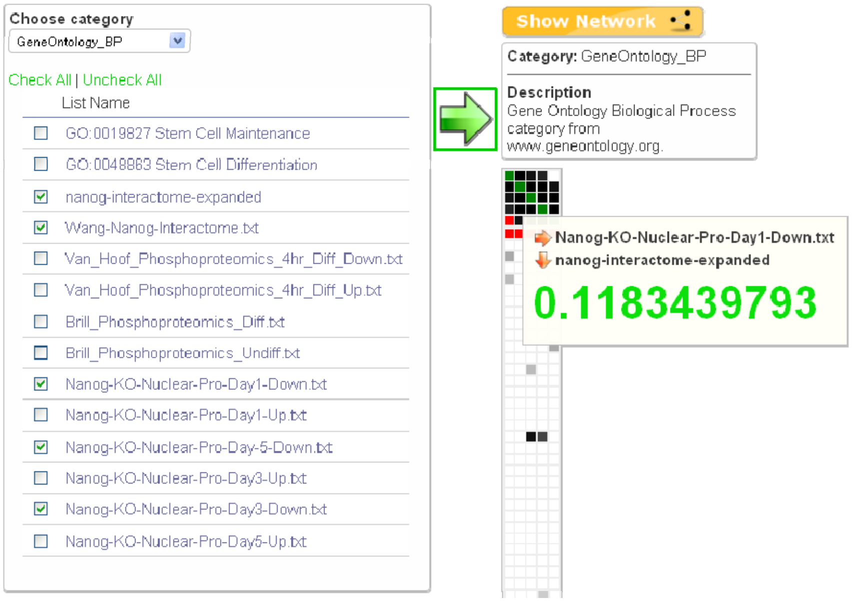

Go to:
http://amp.pharm.mssm.edu/lachmann/upload/overlapmatrix.php

Choose category:
GeneOntology_BP

Select:
Nanog-Interactome-Expanded
Wang-Nanog-Interactome
Nanog-KO-Nuclear-Pro-Day1-Down
Nanog-KO-Nuclear-Pro-Day3-Down
Nanog-KO-Nuclear-Pro-Day5-Down

Press green arrow

The selected lists significantly overlap with each other except for the Nanog-interactome-expanded and the Nanog-KO-Nuclear-Pro-Day1-Down. This can be explained but what was reported in the Lu et al. study, stating that the Nanog iteractome gradually collapses after Nanog knock-down, so at day 1 we expect less overlap as compared with days 3 or 5.

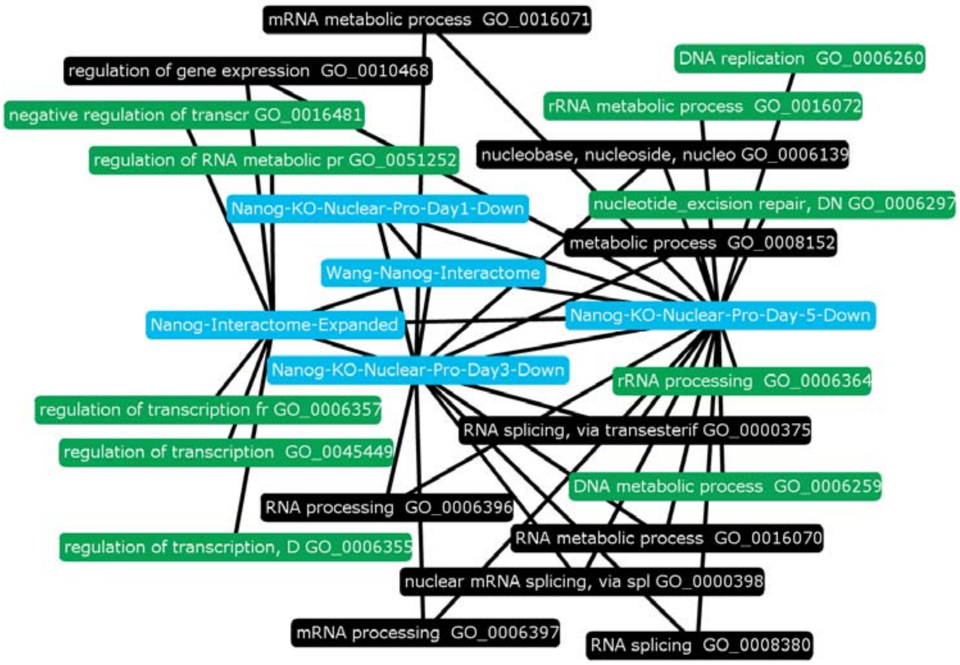

Gene Ontology Biological Process Analysis confirms the high overlap among the Wang et al. interactome and Lu et al. proteins that decrease after differentiation. It suggests that proteins identified by Lu et al. to be down regulated after 5 days are strong candidates to play a role in the self-renewal machinery and be a part of the Nanog interactome. It also points to enrichment in RNA and DNA metabolic processes involving proteins that play a role in the self-renewal network and down regulated after early differentiation.

To further attempt to identify proteins that potentially should be added to the Nanog interactome based on the Lu et al. results we can cross-reference which proteins were added to the Nanog interactome when it was expanded and overlapped with the Lu et al.-Day5-Down-List. Specifically we found that EED, JARID1B, PNO1, SMARCA5 and UTF1 are identified from such cross-reference analysis. These candidates should be further validated as bona-fide self-renewal components. EED, for example, is already known to play such a role.

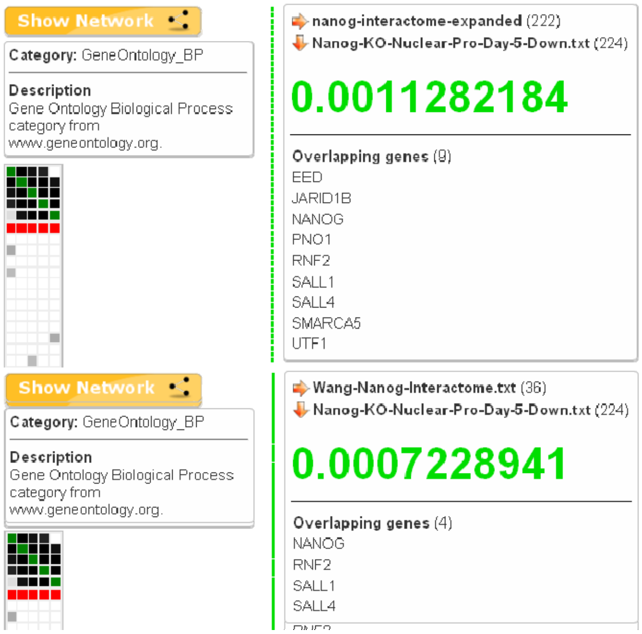

Conclusions

In this case study we have just explored the tip of the iceberg of the proteomics datasets we collected and integrated. We are aware that there may be better ways to filter and extract the data, and currently Lists2Networks does not take into account quantitative changes in expression levels. Our aim in this Case Study is to only demo Lists2Networks for a useful application, namely, integrating proteomics studies applied to embryonic stem cells. However, our hasty analysis already provides stem-cells-biologists food for thoughts, several ideas for further functional experimentation were quickly generated. We showed how, for proteomics and phosphoproteomics, the kinase enrichment, pathway enrichment and GO enrichment analyses can be insightful. For microarray gene expression and other RNA/DNA and chromatin studies the promoter and chip-enrichment analyses may be more appropriate. While other tools for integrative analyses may provide similar functionalities, Lists2Networks makes such analyses more accessible to novice users. Our ability to expand lists, perform lists operations, extract lists from GeneRifs and GO, as well as our newly created gene-list libraries (i.e. KEA and ChIP-x libraries) makes Lists2Networks a great data analysis platform for genome-wide studies.

References

1. Lu, R., et al., *Systems-level dynamic analyses of fate change in murine embryonic stem cells.* Nature, 2009. 462(7271): p. 358-362.
2. Brill, L.M., et al., *Phosphoproteomic Analysis of Human Embryonic Stem Cells.* Cell Stem Cell, 2009. 5(2): p. 204-213.
3. Van Hoof, D., et al., *Phosphorylation Dynamics during Early Differentiation of Human Embryonic Stem Cells.* Cell Stem Cell, 2009. 5(2): p. 214-226.
4. Wang, J., et al., *A protein interaction network for pluripotency of embryonic stem cells.* Nature, 2006. 444(7117): p. 364-368.
